# Sleep-disordered breathing: Aids to diagnosis without a polysomnogram

**DOI:** 10.7196/AJTCCM.2018.v24i4.223

**Published:** 2018-12-20

**Authors:** A Peter

**Affiliations:** Division of Pulmonology, Chris Hani Baragwanath Academic Hospital, Johannesburg, South Africa

**Keywords:** medicine, pulmonology, obesity

## Abstract

The South African population is suffering from an obesity epidemic. Sleep-disordered breathing (SDB), which includes obstructive sleep apnoea and obesity hypoventilation syndrome, is closely related to obesity. SDB may have serious health consequences if not asked about when taking a history related to sleep and sleep-deprivation symptoms. Unfortunately, a formal polysomnogram is available to very few patients who need the diagnosis confirmed. However, taking a sleep history, measuring the haemoglobin level and using a much smaller device in the comfort of a patient’s bed can obviate the need for formal polysomnography.

## Background

Obesity has become a global epidemic, not only of developed countries but of Africa too.^[1]^ A previous demographic health survey among South Africans found that 29% of men and 56% of women are overweight or obese.^[2]^ Doctors seldom take a sleep history, because they think that a polysomnogram (PSG) is essential to diagnosing sleep-disordered breathing (SBD). The condition is therefore seldom picked up, and the consequences of neither obstructive sleep apnoea (OSA) nor obesity hypoventilation syndrome (OHS) are explained to patients. Added to this dilemma is the lack of resources to supply continuous positive airway pressure machines to the vast majority of the obese population, who can neither afford a sleep study nor pay for the machine. The present article aims to evaluate a simple questionnaire using information taken from a history and examination, haemoglobin (Hb) levels and the use of home sleeptesting with an ApneaLink device as an aid to diagnosing OSA. The metabolic consequences of SDB will also be outlined. With these simple tools, patient care and education can be enhanced to promote a greater resolve to lose weight.

## The polysomnogram: The current gold standard in diagnosing OSA

The PSG is widely used as the gold standard in establishing SDB, and home sleep-testing devices are not as accurate. A formal PSG, however, is too expensive for most patients who need one, since the cost is between ZAR2 000 and ZAR5 000. It is a scarce resource for patients in the public sector, compared with those in the private sector. Mulgrew *et al*. ^[3]^ demonstrated that combining the Epworth Sleepiness Scale (ESS), a sleep apnoea clinical score (measuring snoring, apnoea, neck circumference and blood pressure), and a home testing device measuring a respiratory disturbance index, showed a 0.94 positive predictive value (94% accuracy) in making the diagnosis without a PSG. Not using a PSG results in a very small overdiagnosis of OSA; however, the benefits of a diagnosis or highly likely diagnosis, and therapy, outweigh the increased sensitivity.^[3]^

## Snoring – prevalence and health risks

A large number of people snore at night. The Wisconsin Sleep Cohort Study involved 1 843 women and 1 670 men, and found that 28% of women and 44% of men were habitual snorers. Among the habitual snorers, 9% of women and 24% of men had OSA.^[4]^ Not all snorers have apnoea, but the vast of majority of OSA patients snore.

Snoring is three times more common in men than women, and the incidence increases in both sexes with ageing.^[5]^ The louder the snoring, the greater the risk of OSA developing. Mild snoring reaches between 40 and 50 decibels (db), moderate between 50 and 60 db and severe above 60 db.^[6]^ As a comparator, a whisper is in the region of 30 db, normal conversation is between 50 and 65 db and a hair dryer and vacuum cleaner each operate at 70 db. Very loud snoring causes disturbed sleep for those who share the room at night.

Snoring without OSA is a risk for carotid artery atherosclerosis, while not affecting the femoral artery.^[7]^ Snoring on its own was not associated with an increased risk of hypertension.^[8]^

## The STOP-BANG questionnaire

A questionnaire has been developed to detect OSA, since PSG is not widely available. A point is allocated to each of the following: snoring, tiredness, observed apnoea, pressure (hypertension), body mass index (BMI), age, neck circumference and gender (STOP-BANG; (Table 1).^[9]^ Standard values used in the STOP-BANG questionnaire are a BMI>35kg/m² , age >50 years, neck circumference >40 cm for females and 42.5 cm for males, and snoring loud enough to be heard through a closed door. A score of ≥3 is considered a high risk for OSA, while <3 confers low risk.

**Table 1:** STOP-BANG questionnaire (1 point per yes answer)

**Sign or symptom**	**Yes**	**No**
Snoring heard through closed door, louder than talking
Tiredness, fatigue, sleepy during the day
Observed apnoea
Pressure (hypertension)
Body mass index >35 kg/m²
Age >50 years
Neck circumference >40 cm in females, >42.5 cm males
Male sex

The questionnaire shows high sensitivity, with scores of >5, to the maximum of 8, identifying patients at risk for moderate to severe OSA, as confirmed by PSG.^[10]^ A score <2 points excludes a risk of OSA. The specificity of this questionnaire, however, remains low, allowing many false positives, particularly for detecting mild sleep apnoea. Every hypertensive male >50 years old who snores immediately scores 3 in the questionnaire, and is seen as at risk of mild OSA. Harris *et al*.,^[11]^ in a study that used PSG, found that of 110 obese patients showing moderate OSA, a STOP-BANG questionnaire score of ≥4 showed a sensitivity of 85%, but a specificity of 44%. A low specificity makes the test an unreliable diagnostic tool; however, the high sensitivity confers good screening ability.

## The Epworth Sleepiness Scale

Being tired is a subjective feeling. To better answer the second question in the STOP-BANG questionnaire regarding tiredness, the Epworth Sleepiness Scale (ESS; Table 2)^[12]^ attempts to alert both clinician and patient to a degree of tiredness that may well relate to SDB robbing one of restorative sleep.

**Table 2:** Epworth Sleepiness Scale (ESS)*

****	**Likelihood of dozing (point score)**			
**Situation**	**Never (0)**	**Slight (1)**	**Moderate (2)**	**High (3)**
Sitting reading
Watching television
Sitting inactive in a public space
Passenger in a car for 1 hour
Lying down in the afternoon
Sitting talking to someone
Sitting quietly after lunch (no alcohol)
Sitting in a car stopped for a few minutes in traffic

*A score of up to 6 points indicates that enough sleep is being obtained (4 - 8 points = normal). A survey report^[13]^ found that >30% of working people in the USA sleep <6 hours. African ethnicity is associated with higher sleep deprivation, as is night-shift work. A score between 9 and 15 points signals the need to seek medical advice, and >16 points represents a dangerous risk to health. ESS alone is not sufficient to make a diagnosis of OSA.^[14]^

## Improving the poor specificity of the STOP-BANG questionnaire 

### ApneaLink device

**Fig. 1:**
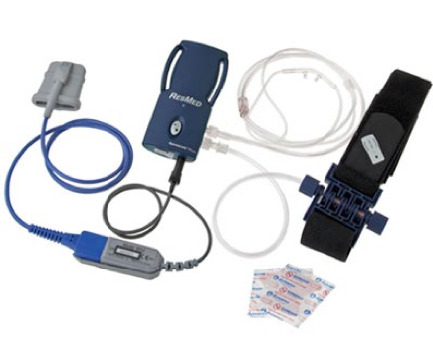
ApneaLink device.

**Fig. 2:**
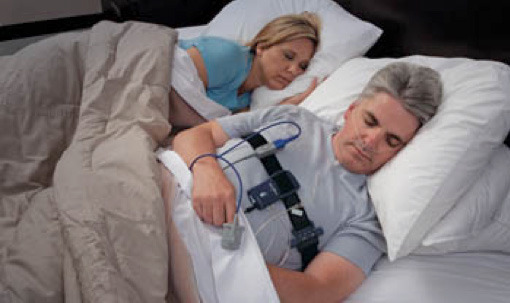
ApneaLink Plus applied to the patient

If a STOP-BANG questionnaire that has a score of ≥4 is coupled with readings from an ApneaLink device (available in South Africa), which measures pulse oximetry, pulse, respiration, apnoeas and hypopnea, then the specificity of the STOP-BANG score is improved. Using an Apnoea Hypopnoea Index (AHI) score of ≥15 raised the specificity from 44% to 89%; if hypertension was present, it rose to 89%; if the neck circumference was >40 cm, it was 92%, and for males 93%. High sensitivities are obtained in all three instances.^[11]^ Compared with PSG, ApneaLink recordings have 100% specificity, but 54% sensitivity, if using an AHI of 15.^[15]^ The high specificity augments the STOP-BANG score. The ApneaLink device is small, and does not require an hour to set up, as does a formal PSG, and it can be used easily at home (Fig. 1). Nasal cannulae are inserted, and the small device is strapped to the chest wall, with a pulse oximeter probe on the finger (Fig. 2). The current price for all components, as well as the software programme that analyses the sleep study, is ZAR14 090, discounted for state institutions, while private purchase is around ZAR17 000 . The only consumable is the nasal cannulae tubing. Using the device multiple times on inpatients in state hospitals, or provided to private patients, is a more economical sleep study method than using the formal PSG in a sleep laboratory.

### Haemoglobin levels

Chung *et al*. ^[16]^ analysed 383 patients with proven OSA, and evaluated the use of a Hb level. They found that Hb levels of ≥16g/dL for men, and ≥15 g/dL for women, when added to a STOP-BANG questionnaire score of ≥3 points, improved the specificity from 35% to 94% for all sleep apnoea, and from 24% to 80% for severe OSA.

Using either the ApneaLink device or an Hb level improves the poor specificity levels of STOP-BANG questionnaire scores between 3 and 5. A STOP-BANG score ≥6 points on its own showed 82% specificity for OSA as measured by PSG.

## SDB in the non-obese

Non-obese individuals may also have SDB, but without the doctor being alerted by obesity, and therefore not taking a patient’s history of sleep, the non-obese sleep-disordered patient can be missed. In a study by Cadavid,^[17]^ 17% of 611 patients referred for OSA assessment were non-obese, and half of these non-obese patients proved to have OSA. The greatest predictors of OSA in the non-obese person proved to be age and gender, rather than sleepiness and ethnic origin. A 1-point increase in BMI signaled an 8% higher odds risk, while being male increased the odds 11.7 fold and a 10-year age increase resulted in a 44% increase in the odds of OSA in the non-obese patient.

## Health risks of OSA

**Fig. 3:**
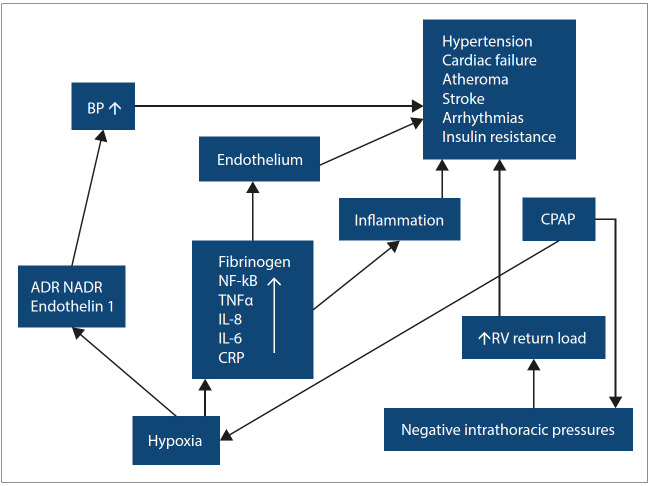
Metabolic consequences of sleep-disordered breathing and the role of continuous positive airway pressure (CPAP). BP = blood pressure (N)ADR = (nanoparticle) adriamycin Nf-kB = nuclear factor kappa-light-chain-enhancer of activated B cells TNF = tumour necrosis factor IL = interleukin CRP = C-reactive protein RV = right ventricular

The pathophysiology of SDB described in Fig. 3 is adapted from an article by Bradley and Floras.^[18]^ The two main drivers are hypoxia and increased negative intrathoracic pressures generated when breathing against an obstructed airway. Continuous positive airway pressure (CPAP) both addresses hypoxia and, by keeping the airway open, decreases the raised negative intrathoracic pressures by opening the airway and releasing the negative intrathoracic pressure so that the lung is able to expand with incoming air. The sympathetic pathway and inflammatory cytokine generation, both affecting the endothelium and causing inflammation, together with increased venous return to the right side of the heart, results in an increased risk of hypertension, cardiac failure, atheroma, stroke arrhythmias and insulin resistance.

The Sleep Heart Health Study^[19]^ established an association between OSA and diabetes. Pallayova *et al*. ^[20]^ demonstrated that in normal glucose metabolism, patients with severe obesity and moderate to severe OSA, decreased levels of pancreatic beta-cell functioning, together with increased insulin resistance, were found. Both are causal in the development of diabetes. Raised tumour necrosis factor a (TNFa) and cytokine interleukin –6 (IL-6) levels correlated with OSA-related oxyhaemoglobin desaturations.

It is controversial whether CPAP can mitigate the development or acceleration of diabetes, and evidence suggests that it may not.^[20]^

## The difference between OSA and OHS

Characteristic findings in OHS are a raised serum bicarbonate level, and a raised CO_2_ of ≥45 mmHg in an arterial blood gas analysis, which are absent in pure OSA. Pulmonary hypertension exists in 15% of OSA alone, while the incidence is between 60% and 88% in OHS, and similarly, the risk of intensive care unit (ICU) admission in OSA alone is 6%, but 40% in OHS.^[21]^ Mortality for patients with OSA admitted to hospital is 9%, compared with 23% for those with OHS.^[21]^ OHS therefore confers greater health risks, and signals a dire need for weight reduction. Ten to 20% of the OSA population will have OHS. African and Asian ethnicity confers a higher risk of OHS, owing to cephalometric differences in cranial structure, and consequent airway diameters, in addition to obesity.^[21]^

## Leptin

Why only a third of very obese patients develop OHS is not clear.^[22]^ Leptin is a hormone produced on chromosome 7 by adipocytes.^[23]^ Leptin suppresses appetite, increases energy expenditure and stimulates the respiratory centre. Leptin resistance in obese people leads to increased food intake, and the inability to increase tidal volume and respiratory rate to cope with the increased work of breathing associated with obesity. Hypercapnia in OHS is postulated to be a cause of insufficient drive on the respiratory centre caused by leptin resistance, or low leptin levels. Leptin resistance is an acquired defect. Leptin acts on the hypothalamus to initiate satiety. Sustained overeating causes high levels of leptin in the cerebrospinal fluid, which leads to the development of hypothalamic receptor resistance. The hypothalamic receptors then fail to initiate satiety. Leptin resistance may also arise from mutations, causing resistance in downstream leptin regulator proteins.^[24]^

## Conclusion

So what can we offer a patient if a PSG is not feasible or available? We should take a sleep history, and look for symptoms of sleep deprivation, either using the STOP-BANG questionnaire or the ESS. Measuring a Hb and PaCO_2_ in patients who have a BMI > 30kg/m² provides clues to the metabolic consequences of hypoxia and OHS (the raised PaCO_2_ ). Using a small device to record an AHI index in the comfort of a patient’s home or hospital bed further establishes a diagnosis of SDB. An electrocardiograph that shows signs of pulmonary hypertension when SDB is present, and other causes have been excluded, is more likely in OHS than OSA, the former of which carries a worse prognosis. Since other illnesses (hypertension, diabetes, cardiovascular disease) are associated with SDB, education about the much wider implications of merely snoring may motivate patients to lose weight.
